# In silico and in vitro analysis of a rational mutation in gIII signal peptide and its effects on periplasmic expression of rhGH in *E. coli*

**DOI:** 10.1007/s00203-022-03193-1

**Published:** 2022-08-24

**Authors:** Fahimeh Ghasemi, Hamed Zare, Alireza Zomorodipour, Maryam Hosseinzade Shirzeyli, Marek Kieliszek

**Affiliations:** 1 Cellular and Molecular Research Center, Birjand University of Medical Sciences, Birjand, Iran; 2 Department of Medical Biotechnology, Faculty of Medicine, Birjand University of Medical Sciences, Birjand, Iran; 3 Pharmaceutical Sciences and Cosmetic Products Research Center, Kerman University of Medical Sciences, Kerman, Iran; 4 Department of Molecular Medicine, Institute of Medical Biotechnology, National Institute of Genetic Engineering and Biotechnology (NIGEB), P.O. Box 14965/161, Tehran, Iran; 5 Department of Anatomical Sciences, School of Medicine, Shahid Beheshti University of Medical Sciences, Tehran, Iran; 6 Department of Food Biotechnology and Microbiology, Institute of Food Sciences, Warsaw University of Life Sciences, Nowoursynowska 159 C, 02-776 Warsaw, Poland

**Keywords:** rhGH, Signal peptide, gIII, *E. coli*, Mutagenesis, Bioinformatics

## Abstract

The secretion efficiency of a heterologous protein in *E. coli* is mainly dictated by the N-terminal signal peptide fused to the desired protein. In this study, we aimed to select and introduce mutations into the – 1, – 2 and – 3 positions of the gIII signal peptide (originated from filamentous phage fd Gene III) fused to the N-terminus of the human growth hormone (hGH), and study its effect on the secretion efficiency of the recombinant hGH into the periplasmic space of *E. coli* Top10. Bioinformatics software such as SignalP-5.0 and PrediSi were employed to predict the effects of the mutations on the secretion efficiency of the recombinant hGH. Site-directed mutagenesis was applied to introduce the desired mutations into the C-terminus of the gIII signal peptide. The periplasmic expression and the secretion efficiency of the recombinant hGH using the native and mutant gIII signal peptides were compared in *E. coli* Top10 under the control of araBAD promoter. Our results from bioinformatics analysis indicated that the mutant gIII signal peptide was more potent than the native one for secretion of the recombinant hGH in *E. coli*. While our experimental results revealed that the mutation had no effect on hGH secretion. This result points to the importance of experimental validation of bioinformatics predictions.

## Introduction

Human growth hormone is a 191-residue polypeptide with a molecular weight of 22 kDa that is produced in the anterior pituitary gland (Rezaei and Zarkesh-Esfahani 2012; Ghavim et al. 2017). With a broad range of biological activities, hGH is used to treat a variety of diseases including growth hormone deficiency, chronic renal failure, and Turner and HIV wasting syndromes (Soares et al. 2003; Rezaei and Zarkesh-Esfahani 2012). Furthermore, its potential to treat bone fractures, skin burns, and bleeding ulcers has been suggested (Soares et al. 2003; Song et al. 2017; Cristóbal et al. 2019).

The hGH is a non-glycosylated protein; therefore, prokaryotic expression systems such as *Escherichia coli* (*E. coli*) are the best choice for its production (Song et al. 2017). High-level expression of recombinant proteins in *E. coli* has been achieved by some optimizations in gene transcription and mRNA translation levels (Makrides 1996; Schumann and Ferreira 2004). However, high-level heterologous gene expression results in the accumulation of recombinant proteins in the cytoplasm of *E. coli* as inclusion bodies which demands several unfolding and refolding steps to acquire proteins with native conformation and correct disulfide bonds formation (Soares et al. 2003; Rosano and Ceccarelli 2014). Moreover, the N-terminal formyl-methionine (fMet) of cytoplasmic proteins can contribute to the development of neutralizing antibodies in patients treated with these therapeutic recombinant proteins (Ghasemi et al. 2004; Siew and Zhang 2021). An option for the production of soluble heterologous proteins in *E. coli* is targeting the recombinant proteins to the periplasmic space using appropriate signal peptides. (Soares et al. 2003; Han et al. 2017). Periplasmic expression of recombinant proteins in *E. coli* has several advantages over cytoplasmic expression. The oxidative condition of the periplasmic space is suitable for proper disulfide bonds formation; therefore, facilitates proper folding of the desired proteins (De Marco 2009; Kleiner-Grote et al. 2018). In addition, correctly folded proteins can be easily purified due to the low amount of undesirable proteins in periplasmic space of the *E. coli* (Ma et al. 2020). The secretion of the recombinant protein into periplasmic space can be achieved by linking a signal peptide to the N-terminus of the proteins (Rosano and Ceccarelli 2014; Pechsrichuang et al. 2016). The signal peptides that direct proteins to the SecB secretory pathway are generally composed of 15–30 amino acids, which are divided into three regions: a positively charged N-terminal region (n-region), which is necessary for association of the signal peptides with the negatively charged membrane, a central hydrophobic region (h-region) that forms an α-helix structure and associates with SecA, and a polar C-terminal region (c-region), with a β-turn conformation, identifies the signal peptide cleavage site (Karamyshev et al. 1998; Zhou et al. 2016; Selas Castiñeiras et al. 2018). It has been proved that the amino acid sequence of a signal peptide determines the efficacy of the translocation of a secretory protein across the inner membrane into the periplasmic space (Zhou et al. 2016; Selas Castiñeiras et al. 2018). The residues at positions – 1 to – 3 (with respect to cleavage site) at c-region has highly conserved with “A–X–A” pattern (where X can be any amino acid). This motif is recognized and cleaved off by the membrane bounded signal peptidase I (Selas Castiñeiras et al. 2018). The residues at positions – 1 and – 3 must be small and uncharged. After translocation of the secretory protein into the periplasmic space, a membrane-bound signal peptidase cleaves off the signal peptide and the mature protein with the correct N-terminal end releases into the periplasmic space of the *E. coli* (Crane and Randall 2017).

Previously, we constructed a recombinant plasmid expressing gIII-hGH preprotein under the control of araBAD promoter. Our results indicated that the gIII-hGH preprotein overexpressed and accumulated in the cytoplasm and only small portion of it was processed and secreted into the periplasmic space of the *E. coli* in the form of mature hGH (Ghasemi et al. 2004).

In the current work, in order to create a more compatible signal peptide that would increase hGH processing and secretion into the periplasmic space, the c-region of the gIII signal peptide, corresponding to – 3, – 2 and – 1 residues) was redesigned and its properties were examined utilizing bioinformatics programs. The secretion efficiency of the new construct was compared with that of the native one. The experimental results showed that despite bioinformatics prediction, no increase in the secretion efficiency of the mutant construct was achieved.

## Materials and methods

### Selection of the mutation at c-region of the gIII signal peptide

Based on the literature and previously described prokaryotic signal peptides, the AMA sequence was selected to introduced into the c-region of the gIII signal peptide to replace SHS sequence.

### In silico analysis

SignalP-5.0 (http://www.cbs.dtu.dk/services/SignalP/) (Nielsen et al. 1999) and PrediSi (http://www.predisi.de) (Hiller et al. 2004), two neural network-based programs, were employed to predict the signal peptide sequences and their cleavage sites in gram negative bacteria. For this purpose, the first 70 residues from the N-terminus of each preprotein (Table 1) were submitted to the signal peptide prediction software to calculate the cleavage probability and signal peptide likelihood. The PHD online server (Rost et al. 1994) was also used to predict the secondary structures of the native and mutant signal peptides. The hydrophobicity of the signal peptides was analyzed using algorithm developed by kyte and Doolittle (Kyte and Doolittle 1982). To calculate hydrophobicity, 40 residues from N-terminus of both native and mutant preproteins were submitted to the protscale online server (https://web.expasy.org/protscale/).

**Table 1:** The first 70 residues from N-terminus of the native and mutant gIII signal peptides

Propeptide name	Amino acid sequence
Native gIII propeptide	MKKLLFAIPLVVPFY**SHS**FPTIPLSRLFDNAMLRAHRLH QLAFDTYQEFEEAYIPKEQKYSFLQNPQTSL
gIIIamq propeptide	MKKLLFAIPLVVPFY**AMA**FPTIPLSRLFDNAMLRAHRLH QLAFDTYQEFEEAYIPKEQKYSFLQNPQTSL

### Bacterial strains, plasmids and primers

Top10 strain of *E. coli* was employed as a host for the cloning steps and expression of hGH. Plasmid pBAD/gIII (Invitrogen, USA), equipped with an L-arabinose inducible promoter (araBAD_)_ and the gIII signal sequence was used for periplasmic expression of hGH in *E. coli*. Previously constructed recombinant plasmid, pBAD/gIII-hGH, harboring hGH cDNA (Ghasemi et al. 2004), was used as a template for PCR-based site-directed mutagenesis of the gIII and amplification of the hGH nucleotide sequence. The primers sequences that were used for site-directed mutagenesis are listed in Table 2.

**Table 2:** The primers used for mutagenesis and amplification of the coding sequences of the gIII-hGH preproteins

Primers names	Primer’s nucleotide sequences (5´ → 3´)	Restriction site
gIII-F1	TTCGGATCCTACCTGACGC	BamHI
GH-start	TTCCCAACTATACCACTACT	–
GH-gIII	GATAGTGGTATAGTTGGGAAGCTATGGCTATAGAACGG	–
GH-stop	CCGGAATTCCTATTAGAAGCCACAGCTGCCC	EcoRI

The restriction sites are underlined

### Media, enzymes and chemicals

*E. coli* was grown in Luria–Bertani (LB) medium containing 10 g/L Bacto-tryptone, 5 g/L.

Bacto yeast extract, and 10 g/L NaCl with the pH adjusted to 7.0. The culture media were supplemented with ampicillin (Roche) with a final concentration of 100 µg/mL when required to maintain selective pressure. Restriction enzymes *Eco*RI, *Bam*HI, and T4 DNA ligase were all purchased from the Thermofisher Scientific (USA). Polyclonal rabbit antiserum against hGH was prepared kindly by Dr. Daliri in NIGEB, Iran. Immuno-reactive bands were detected using horse-radish peroxidase-conjugated goat anti-rabbit antibody (Abcam, UK).

### Sire-directed mutagenesis

Site-directed mutagenesis based on the overlap extension PCR (SOEing PCR) was applied to introduced mutations into the gIII signal sequence. Two primer pairs (given in Table 2) were designed to modify the last three residues of the gIII signal peptide (c-region) from S–H–S to A–M–A, and produce a novel gIIIama signal peptide. Meanwhile, hGH cDNA was linked to the 3̓ end of the gIIIama nucleotide sequence. The SOEing PCR consisted of the following three steps: first, a 155 bp fragment composed of gIII signal peptide and a portion of the upstream araBAD promoter was amplified from the pBAD/gIII plasmid using gIII-F1 and GH-gIII oligonucleotides, respectively, as forward and revers primers. The forward primer was equipped with a *Bam*HI restriction site at the 5´ end and reverse primer contained 20 extra nucleotides from 5´ end of the hGH cDNA, as an overlap. In the next step, the hGH sequence was amplified using GH-start and GH-stop as forward and reverse primers, respectively. The reverse primer was equipped with a *Eco*RI restriction site at the 5´ end. The preceding two PCR products were then purifying from agarose gel 1.5% using High Pure PCR Product Purification Kit (Roche, Germany) and used as templates to amplify the entire fragment containing gIII-hGH sequence using gIII-F1 and GH-stop primers.

### Construction of recombinant plasmid

The amplified hybrid sequence encoding for gIIIama-hGH preprotein was then digested with the *Bam*HI and *Eco*RI restriction enzymes and inserted into the identical sites in the pBAD/gIII plasmid to produce recombinant pBAD/ama-hGH plasmid.

### Growth and induction conditions

To prepare a laboratory scale fermentation condition, a 1/100 dilution of an overnight culture with a working volume of 50 mL was prepared in a 250 mL Erlenmeyer flask. When the cell density of bacteria reached 0.5 < OD_600_ < 1.0 at 37 °C, the induction of recombinant bacteria was performed using 1.33 mM of L-arabinose. Based on our previous study (Ghasemi et al 2004) the cells were harvested after 4 and 16 h of induction as lowest and highest hGH periplasmic expression condition. After that, cytoplasmic and periplasmic proteins were collected and analyzed by SDS–polyacrylamide gel electrophoresis (SDS-PAGE) and western blotting techniques.

### Preparations of cytoplasmic and periplasmic proteins

Periplasmic proteins were extracted using osmotic shock method (previously described by Libby et al. 1987) with some modifications. In brief, 1.5 mL of bacterial suspension with OD_600_ = 1 was centrifuged at 15,000× g for 5 min. The pellet was resuspended in 15 µL of ice-cold TES buffer (0.2 M Tris–HCl, 0.5 M EDTA, 0.5 mM sucrose, pH 8.0) and incubated on ice for 20 min while shaking slowly. Then 22.5 µL of ice-cold double-distilled water was added to the suspended pellet and incubated on ice for 30 min. Finally, the cells were centrifuged at 16,000× g for 20 min, and the supernatant and pellet were collected, respectively, as a periplasmic and cytoplasmic fraction.

### SDS-PAGE and western blotting analysis

SDS-PAGE was performed using 5% stacking and 13% resolving gel according to the method described by Laemmli (1970) with slight modifications (Waśko et al. 2012). Electroblotting of the protein bands onto PVDF membrane (Amersham-Pharmacia Biotech-Germany) was carried out using semidry procedure at 86 mA for 2 h, in a transfer buffer containing 25 mM Tris, 192 mM glycine, 20% methanol. The blot was then probed using a rabbit-polyclonal antiserum prepared against hGH and treated with horse-radish peroxidase-conjugated anti-rabbit antibody (Jeiranikhameneh et al. 2017). Finally, the protein bands were visualized using a solution of 4-chloronaphtol as a substrate.

### Quantification of the hGH periplasmic expression

The AlphaEaseFc software was used to estimate the periplasmic expression of the hGH in *E. coli* Top10. The osmotic shock-fluids from *E. coli* grown under different concentration of L-arabinose, as inducer, were subjected for SDS-PAGE and Coomassie brilliant blue staining. Finally, the density of the protein bands equivalent to the human growth hormone protein were estimated using AlphaEaseFc software.

### Statistical analysis

All experiments were carried out in triplicates. The values were expressed as means ± SD. The statistical analyses were performed by *T* test using Prism software. The *p* values less than 0.05 were considered to be statistically significant.

## Results

### Designing a novel gIII-derived signal peptide

**Fig. 1:**
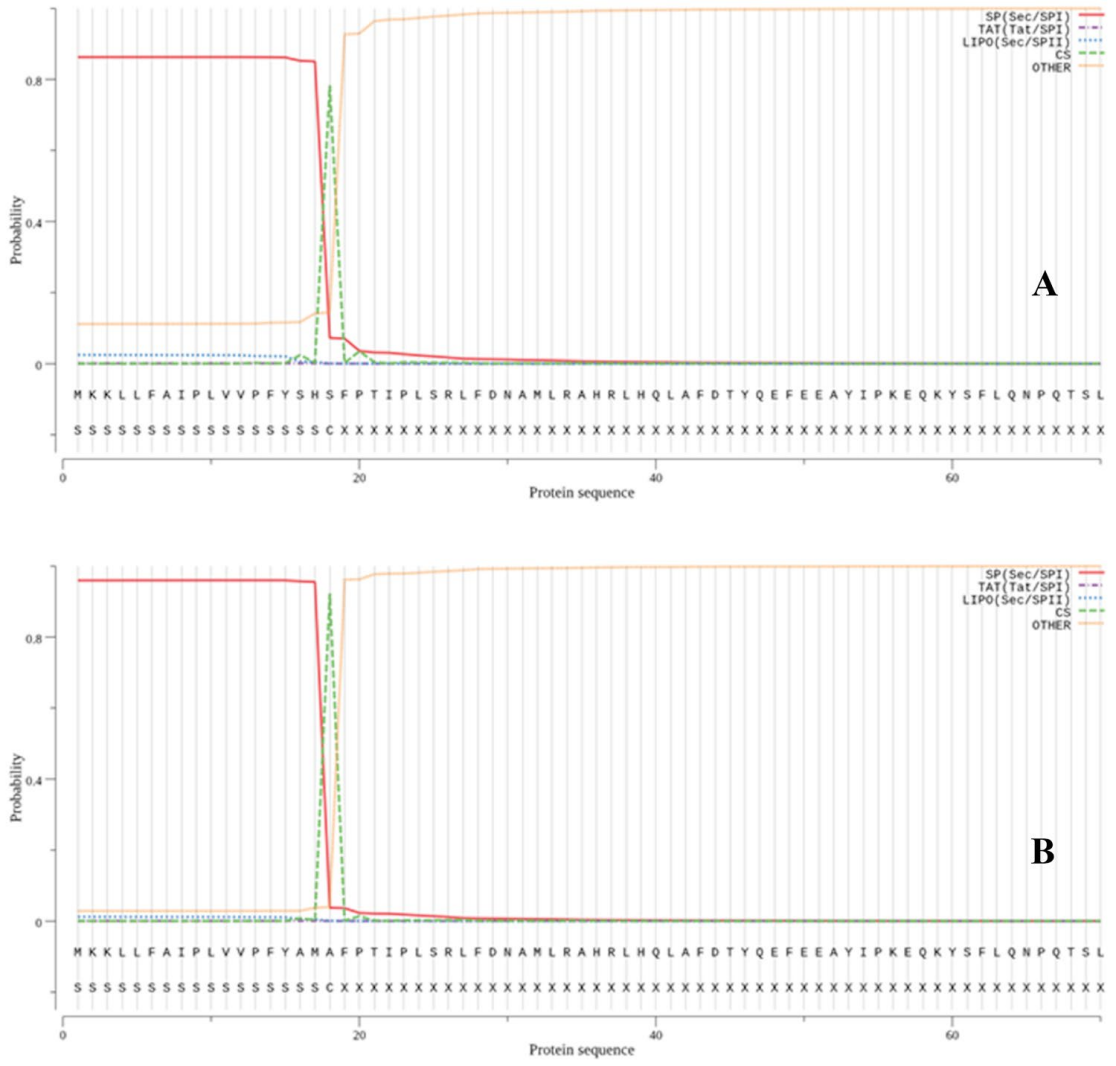
Prediction of the native and mutant preproteins processing using SignalP-5.0. Panel **A** Native gIII-hHG, Panel **B** Mutant gIIIama-hGH preproteins. The first 70 residues from N-terminus of the each preprotein were selected for signal peptide prediction

The first 70 residues from N-terminus of the native and mutant preproteins (gIII-hGH and gIIIama-hGH, respectively) were selected and submitted to the SignalP-5.0 program and the cleavage probability and signal peptide likelihood were calculated (Table 3). Based on the signalP-5.0 analysis, the replacement of the S–H–S by A–M–A at the –1, –2 and –3 positions of the gIII signal peptide significantly increased both the cleavage probability and signal peptide likelihood by 14 and 10%, respectively (Table 3). Furthermore, SignalP-5.0 analysis predicted an accurate cleavage site between residues 18 and 19 for the both native and mutant signal peptides (Fig. 1, green line); therefore, mature hGH with the correct N-terminus was expected to be produced. PrediSi is a software that provides a normalized score on a scale between 0 and 1. A score greater than 0.5 means that the examined sequence very likely contains a signal peptide (Hiller et al. 2004). The PrediSi score of the mutant signal peptides was increased from 0.5738 to 0.8349, showing an improvement in mutant signal peptide property in comparison with the native one (Table 3). The amino acid sequences of the native and mutant signal peptides and their cleavage site have been provided in Table 4.

**Table 3:** In silico prediction of the signal peptide efficiencies using SignalP-5.0, and PrediSi programs, http://www.cbs.dtu.dk/services/SignalP/ and http://www.predisi.de, respectively

Signal peptide name	PrediSi score	SignalP-5.0 prediction		
		Cleavage site position	Cleavage probability	Signal peptide (Sec/SPI)
gIII-hGH	0.5738	18 ↓ 19	0.7830	0.8603
gIIIama-hGH	0.8349	18 ↓ 19	0.9224	0.9572

**Table 4:** The amino acid composition of n- h- and c-regions from native and mutant signal peptides

SP name	SP length	SP regions			
		n-region	h-region	c-region	Cleavage site
gIII	18	MKK (1–3)	LLFAIPLVV (4–12)	PFYSHS (15–18)	SHS-FA
gIIIama	18	MKK	LLFAIPLVV	PFYAMA	AMA-FA

### Prediction of the secondary structure and hydrophobicity of the signal peptides

**Fig. 2:**
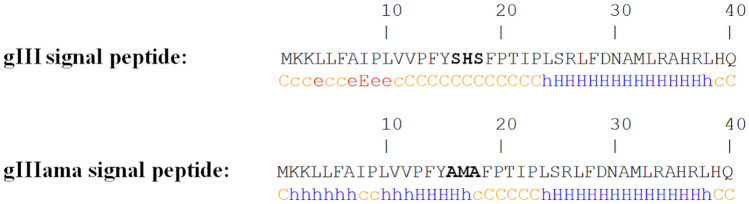
Prediction of the secondary structure of the native and mutant signal peptides using PHD server. The secondary structure of the signal peptide was changed from coil in native signal peptide to α-helix structure in the mutant signal peptide

The secondary structures of the native and mutant signal peptides were predicted using PHD server. As depicted in Fig. 2, the secondary structure of the signal peptide was changed from coil in native signal peptide to α-helix structure in the mutant signal peptide.

**Fig. 3:**
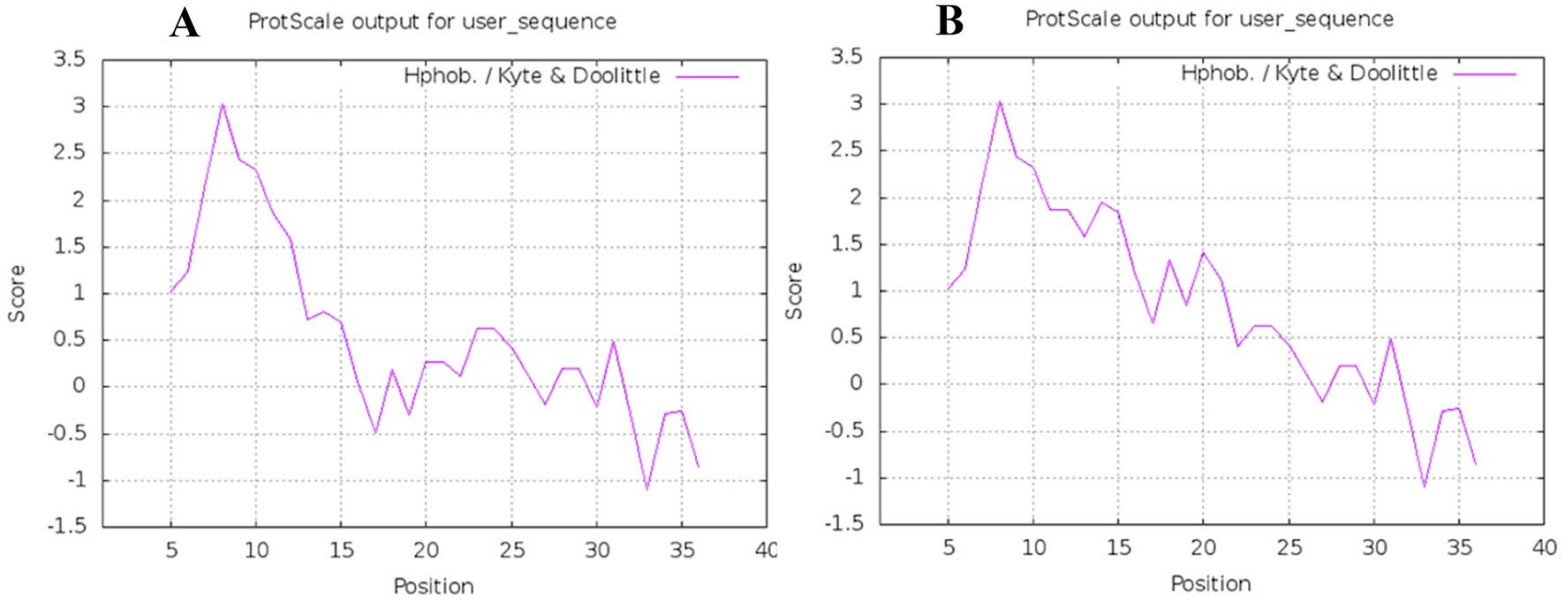
The hydrophobicity of the native and mutant signal peptides. **A** Native, **B** Mutant signal peptides. To calculate hydrophobicity, 40 residues from N-terminus of both native and mutant preproteins were submitted to the protscale online server (https://web.expasy.org/protscale/). The regions with positive values are hydrophobic

The hydrophobicity of the native and mutant signal peptides was compared using algorithm developed by Kyte and Doolittle, showing higher hydrophobicity for the h-region of the mutant gIIIama signal peptide (Fig. 3). The Kyte–Doolittle scale is widely used for detecting hydrophobic regions in proteins. Regions with a positive value are hydrophobic (Kyte and Doolittle 1982)**.**

### Site-directed mutagenesis and construction of recombinant plasmid

**Fig. 4:**
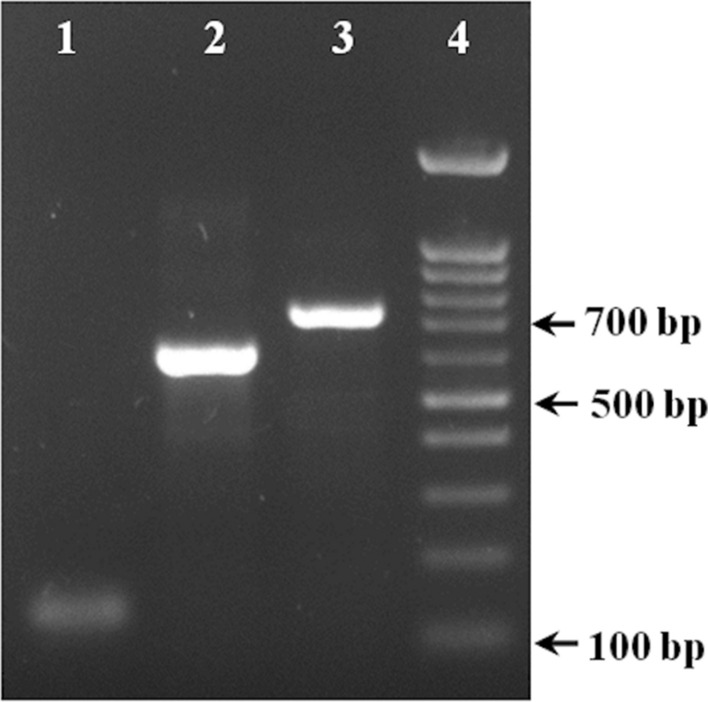
Site-directed mutagenesis of the gIII signal peptide attached to the hGH coding sequence using SOEing PCR. Lane 1: The first PCR product (gIII signal peptide) amplified using gIII-F1 and GH-gIII primers (155 bp); Lane 2: The second PCR product (hGH sequence) amplified using GH-start and GH-stop (590 bp); Lane 3: The final PCR product using gIII-F1 and GH-stop primers (725 bp), Lane 4: DNA ladder (from 100 to 1500 bp)

**Fig. 5:**
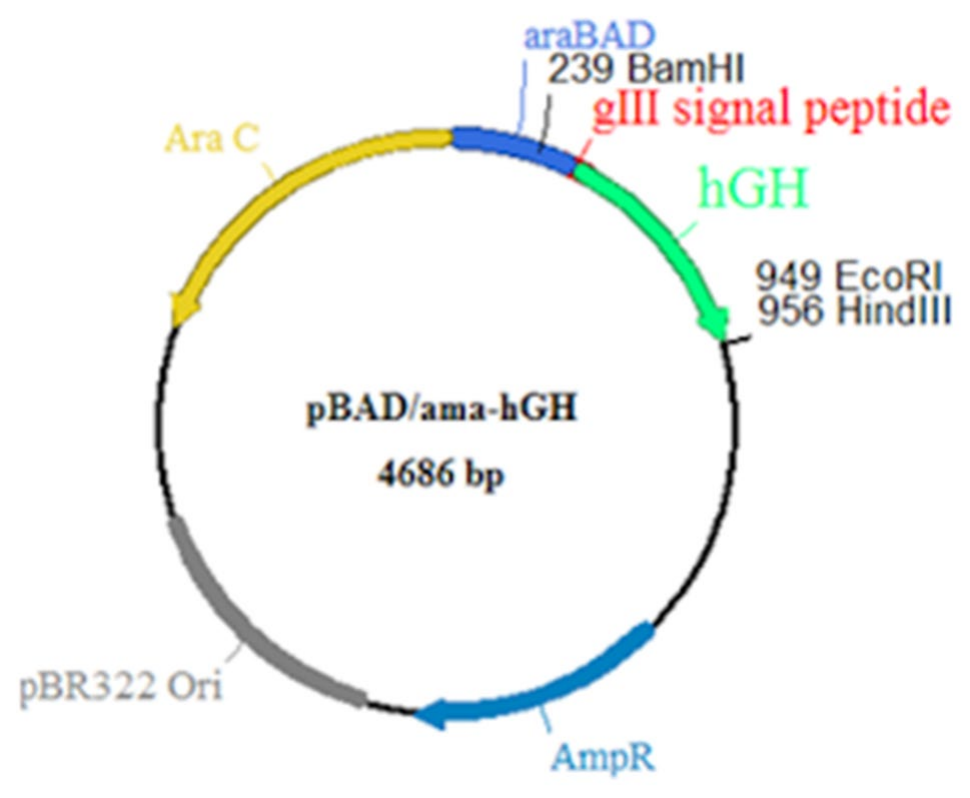
A Schematic map of the pBAD/ama-hGH expression plasmid, showing the major features

To examine the secretion efficiency of the mature hGH using mutant gIIIama signal peptide, PCR-based site-directed mutagenesis was applied to change the three residues located within the C-terminus of the gIII signal peptide from S–H–S to A–M–A (Fig. 2). During the three steps of SOEing PCR, the hGH sequence was attached to the 3̓ end of the mutant gIIIama signal sequence (Fig. 4). The recombinant hybrid sequence, equipped with *Bam*HI and *Eco*RI restriction sites, was cloned into the same sites within the pBAD/gIII expression plasmid to replace gIII signal sequence (Fig. 5).

### Verification of the construction

**Fig. 6:**
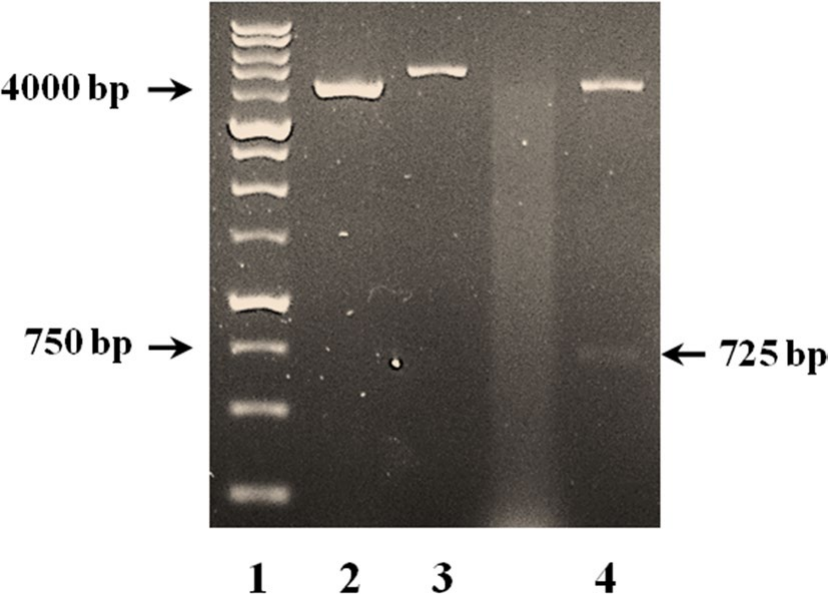
Restriction enzyme digestion of the recombinant construct pBAD/ama-hGH. Lane 1: 1 kb DNA ladder (from 250 to 10,000 bp), Lane 2: pBAD/gIII plasmid digested with *Eco*RI, Lane 3: Recombinant plasmid pBAD/ama-hGH digested with *Eco*RI, Lane 4: Recombinant plasmid pBAD/ama-hGH digested with *Eco*RI and *Bam*HI

To verify accuracy of the recombinant plasmids, PCR tests were performed using the specific primers to amplify the hybrid gIIIama-hGH sequence. The recombinant plasmid was also digested with *Eco*RI and *Bam*HI restriction enzyme to confirm the presence of the gIIIama-hGH fragments (Fig. 6). Finally, the Sanger sequencing was employed for further verification.

### Periplasmic and cytoplasmic expression analysis

**Fig. 7:**
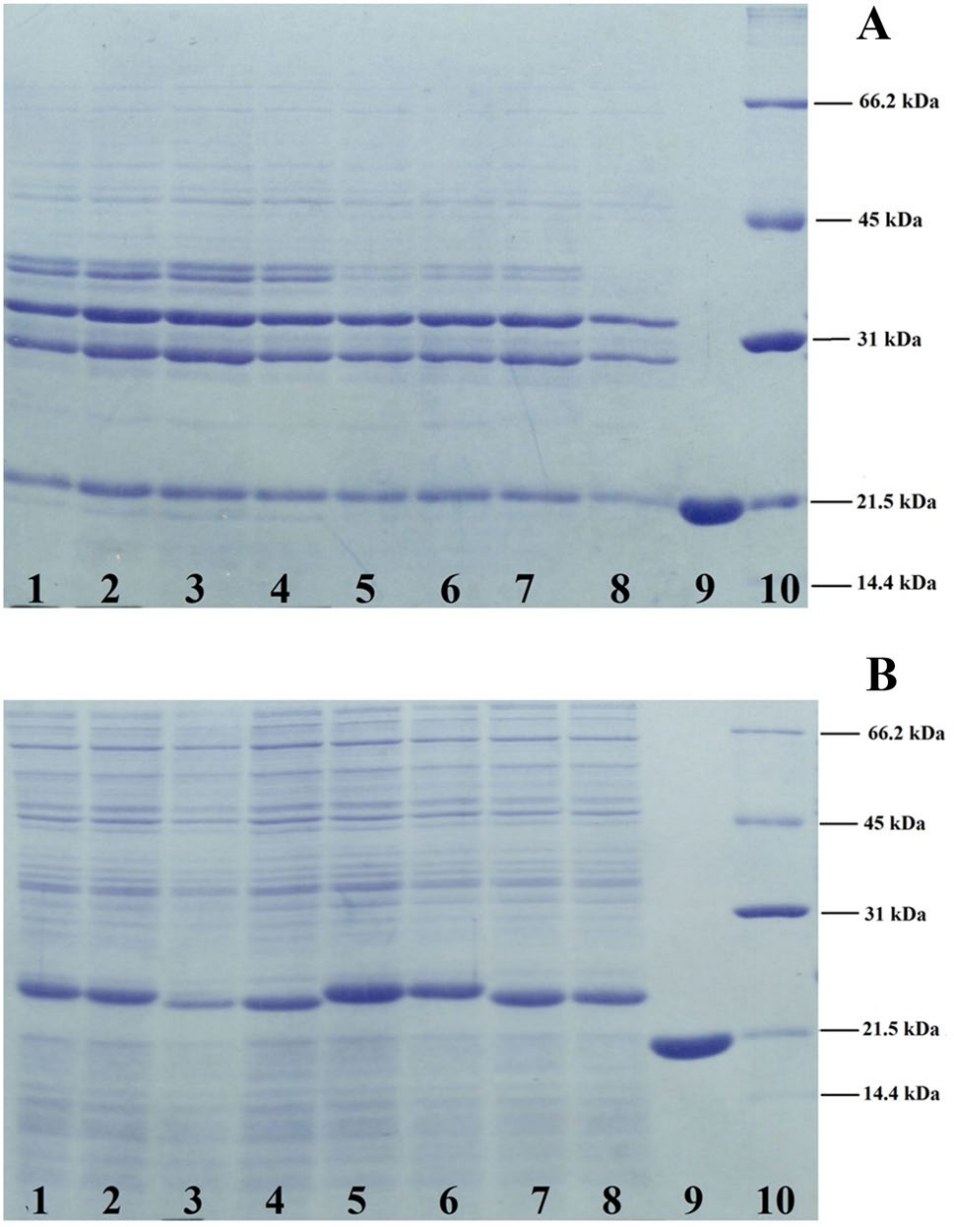
SDS-PAGE analysis of periplasmic (**A**) and cytoplasmic (**B**) proteins from *E. coli* Top10, expressing recombinant hGH after induction with 1.33 mM of L-arabinose. A protein band with molecular weight of 22 kDa is present in the SDS-PAGE of the periplasmic protein extracts. Lanes 1, 2: hGH expression by pBAD/gIII-hGH construct after 4 h of induction. Lanes 3, 4: hGH expression by pBAD/ama-hGH construct after 4 h of induction. Lanes 5, 6: hGH expression by pBAD/gIII-hGH construct after 16 h of induction. Lanes 7, 8: hGH expression by pBAD/ama-hGH construct after 16 h of induction. 9: Standard hGH (Novo Nordisk), 10: Protein ladder (from 14.4 to 66.2 kDa) Broad Range SDS-PAGE Standards

**Fig. 8:**
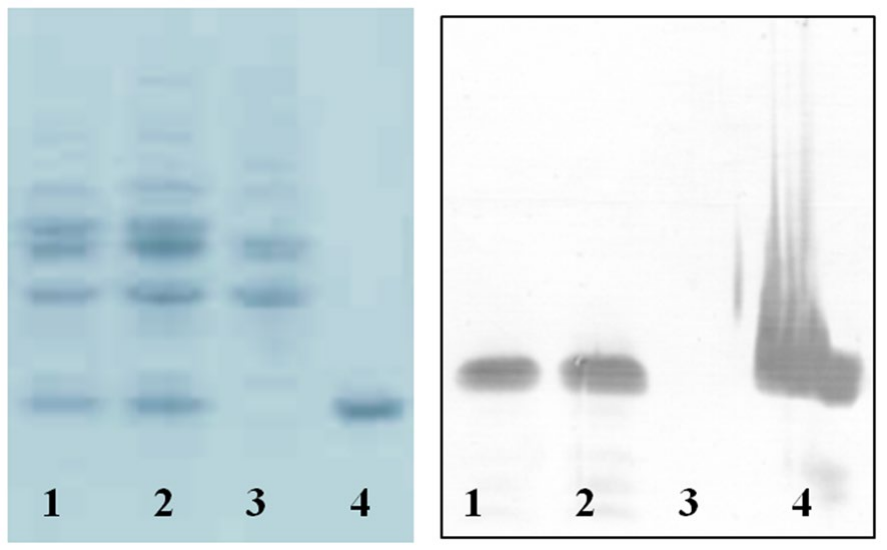
SDS-PAGE (**A**) and Western blotting (**B**) analysis of the recombinant hGH secreted into periplasmic space of the *E. coli* Topt10 after 16 h of induction with 1.33 mM L-arabinose. Lanes 1: mature hGH expressed by pBAD/gIII-hGH construct. Lane 2: mature hGH expressed by the mutant pBAD/ama-hGH construct. 3: periplasmic proteins from uninduced bacteria. Lane 4: Standard hGH (Novo Nordisk)

Top10 strain of *E. coli* was transformed with recombinant plasmids harboring either gIII-hGH or gIIIama-hGH coding sequences and induced with 1.33 mM of L-arabinose. The bacterial suspensions were collected 4 and 16 h after induction. The periplasmic and cytoplasmic proteins were extracted and subjected for SDS-PAGE analysis to visualize the protein bands (Fig. 7). As expected, the mature hGH was present in the periplasmic fraction, while a considerable amount of unprocessed hGH accumulated in the cytoplasm (Fig. 7A and B). The western blotting was then carried out using specific polyclonal anti-hGH antibody to confirm the hGH periplasmic expression (Fig. 8).

### Quantification of the hGH periplasmic expression

**Fig. 9:**
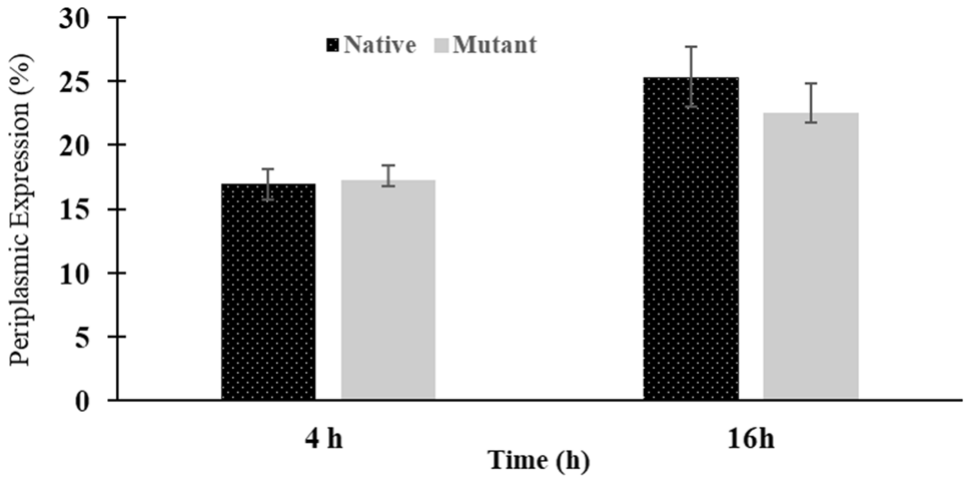
Periplasmic mature hGH expressed by the *E. coli* Top10 harboring either pBAD/gIII-hGH or pBAD/ama-hGH expression vectors, 4 and 16 h after induction by 1.33 mM L-arabinose

The periplasmic proteins were extracted using osmotic shock 4 and 16 h after induction with L-arabinose and subjected for SDS-PAGE. The band equivalent to the mature hGH were quantified using AlphaEaseFc software (Fig. 8). The results demonstrated that in both constructs, the periplasmic expression of the mature hGH greatly increased from 4 to 16 h after induction, but there were no significant differences in the secretion efficiencies of the mature hGH using the native and mutant signal peptides (Fig. 9). The results showed that periplasmic concentration of the mature hGH in the mutant construct was almost the same as the native construct.

## Discussion

Our results from previous studies demonstrated that the recombinant pBAD/gIII-hGH construct harboring gIII-hGH preprotein coding sequence partially secreted mature hGH into periplasmic space of the *E. coli* Top10. Accordingly, the gIII signal peptide could not fully process gIII-hGH preprotein and release mature hGH into the periplasm. A significant amount of the expressed gIII-hGH preprotein accumulated in the cytoplasm and produced inclusion bodies (Ghasemi et al. 2004). In the current work, to improve the secretion efficiency of the gIII-hGH fusion protein, we introduced mutations into the C-terminus of the gIII signal peptide at positions of – 1, – 2 and – 3 (with respect to the cleavage site).

It has been proved that C-terminus of the signal peptides in bacteria is highly conserved and the A–X–A motif is dominated in this region (Selas Castiñeiras et al. 2018). It has been reported that 139 of 151 signal peptides from gram negative bacteria contained Ala at position – 1, while the position – 3 was less conserved and occupied by Ala in 99 signal peptides (Karamyshev et al. 1998). Moreover, in a study conducted by Zamani et al. (2016), 23 different signal peptides, including the natural hGH and 22 prokaryotic signal peptides were evaluated and their efficacies for the secretion of the hGH into periplasmic space of the *E. coli* were predicted using bioinformatics tools such as SignalP (Zamani et al. 2016). Twelve signal peptide had high D score with AXA pattern at c-terminus and the highest D score was related to the PelB with AMA sequence (Zamani et al. 2016).

Therefore, in this study A–M–A sequence was selected to be introduced into the C-terminal end of the gIII signal peptide to replace S–H–S sequence at positions – 1, – 2 and – 3. The new gIII-derived signal peptide was named gIIIama.

First, to predict the accuracy of the cleavage site and efficiency of the signal peptide processing and secretion of the mature hGH, some bioinformatics tools such as SignalP-5.0 and PrediSi was employed. The mutant gIIIama-hGH preprotein sequence was submitted to SignalP-5.0 and PrediSi software and the secretion efficiencies of the mature hGH into in gram negative bacteria was predicted.

The results demonstrated that replacement of the S–H–S by A–M–A at the C-terminus of the gIII signal peptide could improve the secretion. Two scores were calculated using SignalP-5.0 for both native and mutant signal peptides (cleavage probability and signal peptide likelihood). By this mutation, the scores were increased from 0.7830 and 0.8603 to 0.9224 and 0.9572, respectively (Table 3). The scores demonstrated that the mutation improved the cleavage probability and signal peptide likelihood scores by 14 and 10%. Moreover, PrediSi score was increased from 0.57 to 0.83 which indicated in the improvement of the secretion due to the mutation.

It has been revealed that, the conformation and secondary structure of the h- and c-region determine the efficiency of the signal peptide and precise cleavage site (Karamyshev et al. 1998; Pratap and Dikshit 1998). In an ideal signal peptide, the h-region forms an α-helix structure. In this regards, the native and mutant signal sequences were analyzed using PHD secondary structure prediction method (Rost et al. 1994). Our results demonstrated that secondary structure of the h-region was converted from the coil to the α-helix structure, while a coil structure was maintained at cleavage site (Fig. 2). In fact, the mutation resulted in a signal peptide with a more favorable secondary structure at h- and c-region.

The hydrophobicity of the h-region is another factor that influence the efficacy of a signal peptide. A lot of studies have suggested that increasing the hydrophobicity of the h-region in a signal peptide improves protein translocation across the inner membrane in *E. coli* (Zhou et al. 2016). However, a small number of mutagenesis studies have demonstrated that increasing hydrophobicity of the h-region did not affect the protein secretion. Therefore, the hydrophobicity of the native and mutant gIII signal peptides was analyzed using algorithm developed by kyte and Doolittle (Kyte and Doolittle 1982). The results demonstrated that the hydrophobicity in the h-region of the gIII signal peptide increased by mutation (Fig. 3).

Based on the results from in silico analysis and confirmation of suitability of S–H–S replacement by A–M–A, site-directed mutagenesis was applied to replace S–H–S amino acid sequence by A–M–A at the C-terminal region of the gIII signal peptide within – 1, – 2 and – 3 positions. The fusion sequence was then inserted into the pBAD/gIII expression vector under the control of the araBAD promoter in a way to replace gIII signal peptide. The protein expression profiles of the periplasmic and cytoplasmic fractions were prepared using SDS-PAGE after induction of recombinant bacteria with 1.33 mM L-arabinose for 4 and 16 h. The results demonstrated that a considerable amount of native and mutant preproteins accumulated unprocessed in the cytoplasm and only a small portion of the mature hGH was found in the periplasm. Our findings revealed that there were no significant differences in secretion efficiencies of the native and mutant signal peptides. Although in silico analyses indicated that the mutant gIII-derived signal peptide was more potent than the native one for secretion of the hGH in gram negative bacteria, our experimental analysis did not confirm the in silico predictions. In fact, the compatibility of a signal peptide with the protein of interest is a main criterion for efficient secretion of mature protein. Some bioinformatics tools have been developed to predict the compatibility of a signal peptide with the desired protein, but their accuracy is not complete and should be examined experimentally. Other factors such as the rate of recombinant protein translation might also influence the secretion of the heterologous proteins in *E. coli*. It is assumed that, the translation rate must be matched with the capacity of the translocation machinery of the cell, otherwise misfolded preproteins would accumulate in the cytoplasm and would not enter into the secretion pathway (Horga et al. 2018). In our investigation, the overexpression of the hGH might block the translocation machinery and reduce the transport of the preprotein into periplasmic space of *E. coli*. Moreover, studying the effect of only one kind of mutation in the c-region of the gIII signal peptide is insufficient to come to a conclusion. Therefore, we need to do further investigation by introducing more mutations in this region to get the desired results.

## Conclusion

In the current work, based on the bioinformatics analysis, amino acid substitution was introduced into the c-region of the gIII signal peptide to study the effect of the mutation on the secretion of the recombinant hGH into the periplasmic space of the *E. coli*. Although in silico analyses predicted that the mutant gIII signal peptide was better signal peptide than the native one for secretion of the hGH in *E. coli*, our experimental analysis did not confirm the in silico prediction. Bioinformatics tools have been developed to predict the compatibility of a signal peptide with a desired protein, but their accuracy is not complete and should be examined experimentally. Other factors influence the secretion of the recombinant proteins in *E. coli* that need to be tested in vitro.

## Data Availability

Not applicable.
